# Characterization of MultidrugResistant serogroup 19 *Streptococcus pneumoniae* isolated from healthy children below 5 years of age in Indonesia

**DOI:** 10.1099/acmi.0.000680.v4

**Published:** 2024-02-13

**Authors:** Korrie Salsabila, Yayah Winarti, Wisiva Tofriska Paramaiswari, Wisnu Tafroji, Hanifah Fajri Maharani Putri, Wa Ode Dwi Daningrat, I Gusti Ayu Inten Wulandari, Amin Soebandrio, Dodi Safari

**Affiliations:** ^1^​ Eijkman Research Center for Molecular Biology, National Research and Innovation Agency (BRIN), Cibinong, West Java, Indonesia; ^2^​ Master’s Program in Biomedical Sciences, Faculty of Medicine, Universitas Indonesia, Jakarta, Indonesia; ^3^​ Department of Clinical Microbiology, Faculty of Medicine, Universitas Indonesia, Jakarta, Indonesia

**Keywords:** biofilm, gene resistance, multidrug-resistant *Streptococcus pneumoniae* (MDRSP), pilus, sequence type

## Abstract

We investigated the resistance genes, pilus islets, biofilm formation ability and sequence types of multidrug-resistant *Streptococcus pneumoniae* (MDRSP) isolated from healthy children below 5 years of age in Indonesia. In all, 104 archived MDRSP isolates from previous carriage studies in Indonesia in 2016–2019 were screened for the presence of antibiotic resistance genes and the *rrgC* (pilus islet 1) and *pitB* (pilus islet 2) genes. Multilocus sequence typing and biofilm formation were determined by PCR sequencing and the ability of cells to adhere to the walls, respectively. Results have shown that the *mefA*, *ermB* and *tetM* genes were found in 93, 52 and 100 % of MDRSP isolates, respectively. Insertions of arginine, proline and Ile-100–Leu were the most common mutations in the *folA* and *folP* genes. Pilus islets 1 and 2 were discovered in 93 and 82 % of MDRSP isolates, respectively. The MDRSP isolates showed no biofilm formation ability (64 %), and 5 out of 10 strains of MDRSP strains were ST1464. This finding can be used to provide further considerations in implementing and monitoring pneumococcal vaccination in Indonesia.

## Data Summary

All data on characterization of multidrug-resistant serogroup 19 *Streptococcus pneumoniae* are available in Table S1, available in the online version of this article and the PCR condition and primer lists are available in Table S2.

## Introduction

*Streptococcus pneumoniae* is a Gram-positive, lancet-shaped, diplococcus bacteria that is facultatively anaerobic [1]. This bacterium is part of the normal flora in the nasopharynx, although it is able to invade sterile sites of the body to cause infections such as meningitis, bacteraemia and pneumonia [2]. Unfortunately, invasive pneumococcal disease (IPD) has also not been monitored systematically in Indonesia.

Although antibiotics have significantly reduced the mortality rate of pneumococcal infections, the misuse and overuse of antibiotics have caused the development of resistant clones through adaptation mechanisms against antibiotic pressure [3]. Penicillin-non-susceptible *S. pneumoniae* are one of the World Health Organization’s (WHO’s) priority pathogens for which the development of new antibiotics is urgently needed [4]. Macrolides are often used to treat upper respiratory infections and pneumonia caused by *S. pneumoniae*.

Macrolide resistance is mediated by *ermB* and *mefA* genes. *ermB* encodes methyltransferase enzyme, which causes ribosomal methylation to prevent binding of macrolide. Thus, protein synthesis can continue. The *mefA* gene encodes efflux pumps to reduce the accumulation of macrolide within bacterial cells [5]. Another widely used antibiotic to treat pneumococcal infection is sulphametoxazole–trimethoprim (co-trimoxazole). This antibiotic acts as substrate inhibitor for dihydrofolate reductase (DHFR) and dihydropteroate synthase (DHPS), the crucial enzymes for folate biosynthesis in bacteria. Mutations in *folA* and *folP* genes, which encode DHFR and DHPS, respectively, cause co-trimoxazole resistance, since antibiotics fail to recognize and bind to the enzymes [6]. Tetracycline resistance is mediated by *tetM* enzymes, which express proteins that will bind and protect ribosome from tetracycline, so that amonoacyl-tRNA can bind to the ribosome and the translation process can continue [7].

Multidrug-resistant *S. pneumoniae* (MDRSP) are defined as pneumococci that are resistant to at least three classes of antibiotics. MDRSP were found at a prevalence of 59.3 % in Asia with serotypes 19F and 19A being the most dominant [3]. In Malaysia, serotypes 19F and 19A were the most frequent serotypes isolated from blood and cerebrospinal fluid of children <5 years of age [8]. Moreover, 46 % children <5 of age in Lombok, Indonesia carried *S. pneumoniae* and the second most common serotype found was 19F [9]. The highest antibiotic resistance rates among the pneumococcal isolates in Lombok were for the antibiotics tetracycline and co-trimoxazole [9]. Safari *et al*. also reported that pilus types 1 and 2 were found among pneumococcal isolates in Indonesia and that serotypes 19F and 19A were dominant [10]. Taiwan^19F^-14 clone serotypes 19F and 19A were also discovered among penicillin- and macrolide-non-susceptible isolates [11]. In Indonesia, serotypes 19F and 19A are the most frequent serotypes among MDRSP isolates. However, studies regarding the resistance mechanisms, and other factors such as pilus and biofilm formation, that can contribute to the virulence and resistance of MDRSP isolates serotypes 19F and 19A in Indonesia are limited. Thus, this study aims to characterize MDRSP serotypes 19F and 19A in Indonesia, including their resistance genes (macrolide and tetracycline resistance genes; mutation in *folA* and *folP* in co-trimoxazole resistance), pili, biofilm and sequence types (STs).

## Methods

### *S. pneumoniae* isolates

In total, 104 archived MDRSP serotypes 19F (*n*=100) and 19A (*n*=4) isolates were collected from previous nasopharyngeal carriage studies among healthy children below 5 years of age from different regions in Indonesia: Kotabaru, South Kalimantan [12] (2019: 40 isolates 25 isolates); Southwest Sumba, East Nusa Tenggara (2016: 28 isolates) and Gunung Kidul, Yogyakarta (2016: 40 isolates) ([13], under review); and Wakatobi, Southeast Sulawesi (2018: 11 isolates) ([14], under review). The data for *S. pneumoniae* isolates can be seen in Table S1.

### Resistance gene detection and pilus identification

The detection of resistance genes and pilus identification were performed using conventional PCR [15–19]. DNA sequencing was then conducted to identify mutations in the *folA* gene encoding for dihydrofolate reductase (DHFR) and *folP* gene encoding for dihydropteroate synthase (DHPS) in co-trimoxazole resistance. The PCR condition and primer list can be seen in Table S2.

### Biofilm formation

ATCC *Staphylococcus aureus* 25 923 was used as a positive control (strong producer biofilm) and ATCC *Streptococcus agalactiae* 13 813 was used as a negative control (non-producer biofilm) [20]. The tested *S. pneumoniae* isolates and *S. agalactiae* were cultured onto Trypticase Soy Agar II (BD) with 8 % sheep blood. *S. aureus* was streaked onto Tryptic Soy Agar (BD). All isolates were incubated at 37 °C with 5 % CO_2_ for 18–24 h. A bacterial suspension equivalent to 0.5 McFarland was then prepared from the overnight culture in 5 ml Mueller–Hinton Broth (MHB) medium. Then, 30 µl of the 0.5 McFarland bacterial suspension was transferred into 3 ml brain heart infusion (BHI) supplemented with rabbit serum (2.5 ml BHI and 0.5 ml rabbit serum). The suspension was then incubated at 37 °C with 5 % CO_2_ for 5 h until it reached log phase [20].

The bacterial suspension in BHI supplemented with rabbit serum was then adjusted to be equivalent to 0.5 McFarland in 3 ml MHB medium. Then, 1.5 ml of the bacterial suspension was transferred to a microcentrifuge tube and centrifuged at 10 000 r.p.m. for 2 min. The pellets were washed twice with 1 ml phosphate-buffered saline (PBS) 1×. After that, 1.5 ml MHB was added and 5 µl was transferred to 995 µl BHI. One hundred microlitres of the suspension was added into each well of a flat bottom 96-well plate (samples were tested in triplicates) and then incubated at 37 °C with 5 % CO_2_ for 18 h [20].

After incubation, all of the bacterial suspension was discarded. Biofilm that had formed on the bottom of wells was washed twice using 150 µl PBS 1×. Biofilm staining was then performed by incubation with crystal violet 0.5 % for 15 min. The suspension was removed and washed twice using 150 µl PBS 1×. Absolute ethanol 150 µl was added into each well to rinse the stained biofilm and then transferred into a new flat bottom 96-well plate for optical density measurement. Optical density (OD) was measured at 595 nm using the Varioskan (Thermo Fisher Scientific). The biofilm formation index (BFI) was calculated using the following formula: BFI=AB (the average OD of the sample)–CW (the average OD of BHI as blank). The cut-off values were used to determine the biofilm production ability of *S. pneumoniae*. An obtained BFI >0.30 was classified as a strong producer, 0.10–0.30 as a moderate producer and <0.10 as a non-producer [21].

### Multilocus sequence typing (MLST)

MLST was performed for 10 selected MDRSP isolates of serotypes 19F and 19A. Ten isolates were chosen, eight of them were penicillin-resistant *S. pneumoniae* with a high MIC value (>4 µg ml^−1^), while the remaining two were intermediate and susceptible to penicillin. The WKT0041 isolate that was susceptible to penicillin was the only one categorized as a strong biofilm producer in this study. All 10 isolates were resistant to macrolide, tetracycline and co-trimoxazole. A conventional PCR sequencing method was used to detect seven housekeeping genes in *S. pneumoniae* as described previously [22].

DNA sequences from seven housekeeping genes were aligned with the positive controls of each gene. Then fasta format data were submitted to public databases for molecular typing and microbial genome diversity (PubMLST) to obtain allele number for each locus [23]. A combination of the allele number of seven housekeeping genes determined the STs. The obtained STs were further analysed using Based Upon Related Sequence Types (burst) in PubMLST to describe the relatedness of the isolates [23].

## Results

### Genotype and phenotype of MDRSP serotypes 19F and 19A

The antibiotic resistance rate among the MDRSP isolates was identified in the previous study as follows: resistance to tetracycline (99 %; 103/104), co-trimoxazole (95 %; 99/104), erythromycin (91 %; 95/104), chloramphenicol (1 %; 1/104) and penicillin (22 %; 23/104) [12, 13, 13]. The MIC ranges of MDRSP in correlation with the genotypic results in this study can be seen in Table 1. However, the MIC value was only available for 93 of the isolates; the antimicrobial susceptibility testing (AST) for the rest of the 11 isolates were performed through disc diffusion method.

**Table 1.** Genotypes and phenotypes of Multidrug-resistant *S. pneumoniae* isolates

Phenotype	Genotype	n* (%)	Antimicrobial susceptibility profile (µg ml^−1^)					
			Penicillin	Azithromycin	Erythromycin	Tetracycline	Co-trimoxazole	Chloramphenicol
Cotrimoxazole resistance (*folA*)	Asp-92–Ala, Ile-100–Leu	1 (1 %)	≤0.03	>2	>2	>8	>4/76	=4
	Asp-92–Ala, Glu-94–Asp, Ile-100–Leu	92 (99 %)	=1–>4	=2–>2	=2–>2	=8–>8	=4/76–>4/76	=2–=4
Cotrimoxazole resistance (*folP*)	STRPGSSYYVEIE	32 (34 %)	=1–>4	>2	=2–>2	>8	=4/76–>4/76	=2–=4
	STRPRPGSSYVEIE	60 (65 %)	≤0.03–>4	=2–>2	=2–>2	=8–>8	>4/76	=2–=4
	No mutation	1 (1 %)	>4	>2	>2	>8	=4/76	=4
Macrolide resistance	*ermB* (+)	52 (56 %)	≤0.03–>4	>2	>2	=8–>8	=4/76–>4/76	=2–=4
	*mefA* (+)	90 % (97 %)	=1–>4	=2–>2	=2–>2	=8–>8	=4/76–>4/76	=2–=4
	*ermB* (+) and *mefA* (+)	49 (53 %)	=2–>4	>2	>2	=8–>8	=4/76–>4/76	=2–=4
	*ermB* (+) and *mefA* (−)	3 (3 %)	≤0.03–>4	>2	>2	>8	>4/76	=2–=4
	*ermB* (−) and *mefA* (+)	41 (44 %)	=1–>4	=2–>2	=2–>2	>8	=4/76–>4/76	=2–=4
Tetracycline resistance	*tetM*	93 (100 %)	≤0.03–>4	=2–>2	=2–>2	=8–>8	=4/76–>4/76	=2–=4
Pilus	Type 1	90 % (97 %)	=1–>4	=2–>2	=2–>2	=8–>8	=4/76–>4/76	=2–=4
	Type 2	81 (87 %)	≤0.03–>4	=2–>2	=2–>2	=8–>8	=4/76–>4/76	=2–=4
	Type 1 only	12 (13 %)	=2–>4	>2	>2	>8	>4/76	=2–=4
	Type 2 only	3 (3 %)	≤0.03–=4	>2	>2	>8	=4/76–>4/76	=2–=4
	Types 1 and 2	78 (84 %)	=1–>4	=2–>2	=2–>2	=8–>8	=4/76–>4/76	=2–=4
Biofilm formation	Moderate	34 (37 %)	≤0.03–>4	>2	=2–>2	>8	=4/76–>4/76	=2–=4
	Negative	59 (63 %)	=2–>4	=2–>2	=2–>2	=8–>8	=4/76–>4/76	=2–=4

*n=93; 11 isolates were not included in this table because the antimicrobial susceptibility testing was performed by disc diffusion, meaning that MIC values could not be obtained.

All the tested AST broth microdilutions of the study isolates displayed high resistance against tetracycline with MIC the range=8–>8 ng ml^−1^ (100 %; 93/93). Penicillin-resistant *S. pneumoniae* (MIC >4 µg ml^−1^) (14 %; 13/93) carried either *ermB* or *mefA*, possessed the type 1 pilus (*rrgC*) and were dominated by negative biofilm producers. Isolates possessing the Asp-92–Ala and Ile-100–Leu mutations in *folA* (1 %; 1/93) and STRPRPGSSYVEIE variation in *folP* (65 %; 60/93), those that were *ermB*-only-positive (3 %;3/93), and type 1 pilus-only isolates (13 %;12/93), showed high resistance to co-trimoxazole with MICs >4/76 µg ml^−1^. The study isolates were still susceptible to chloramphenicol, with MICs of 2–4 µg ml^−1^. The MDRSP strains also showed resistance to macrolide antibiotics such as azithromycin and erythromycin (89/93). Macrolide resistance with high MICs (2 µg ml^−1^) was found in isolates with the mutations Asp-92–Ala and Ile-100–Leu in *folA*, those that were *ermB*-positive, type 1 or type 2-only pilus isolates, and moderate biofilm producers.

In isolates carrying the *ermB* gene 52 % showed high resistance to macrolides (MIC 2 µg ml^−1^) compared to the isolates that did not possess the *ermB* gene (Table 1). The MDRSP strains carrying only the *mefA* gene were also higher (44 %) compared to isolates with the *ermB* gene alone (3 %). However, the *mefA* gene was associated with less resistance against macrolides. The MDRSP isolates carrying both *ermB* and *mefA* were almost half of the tested isolates (50 %), and resistant against macrolide with high MIC values (>2 µg ml^−1^). There were three isolates that did not carry either *mefA* or *ermB* (2.86 %) and were still susceptible to macrolides. Meanwhile, the *tetM* gene, which encodes the protein that protects ribosomes from tetracycline, was found in all isolates of MDRSP (104/104). The MIC value for tetracycline was also the highest among other tested antibiotics (=8–>8 µg ml^−1^).

The *folA* gene encoding for dihydrofolate reductase (DHFR) and the *folP* gene encoding for dihydrofolate synthase (DHPS) were detected by conventional PCR. The *folA* and *folP* genes were found in all isolates of MDRSP. Sulfamethoxazole resistance was mostly mediated through the repetition of arginine and proline after amino acid 59 in the *folP* gene (STRP ***RP*** GSSYVEIE), as seen in Table 2. Meanwhile the combination of amino acid substitutions Asp-92–Ala, Glu-94-Asp and Ile-100–Leu was the most common substitution at amino acids 92–100 of *folA* (Table 2). *folA* sequences are available at GenBank with accession numbers MW816708–MW816815, and *folP* with accession numbers MW835823–MW835925.

**Table 2.** Mutation of *folA* and *folP* among MDRSP serotype 19F and 19A isolates

*folA* mutation (amino acid 92–100)	*folA* variation	*n* (%)
Substitution	Asp-92–Ala	2 (2)
	Asp-92–Ala, Glu-94–Asp, Ile-100–Leu	96 (92)
	Asp-92–Ala, Ile-100–Leu	5 (5)
	Asp-92–Ser, Glu-94–Asp, Ile-100–Leu	1 (1)
***folP*** **mutation (amino acid 56–67)**	***folP*** **variation**	***n*** **(%)**
3b insertion	STRPGSSY ***Y*** VEIE	37 (36)
6 bp insertion	STRPGSSY ***SY*** VEIE	2 (2)
	STRP ***RP*** GSSYVEIE	63 (61)
No mutation	STRPGSSYVEIE	2 (2)

### Pilus identification

Pili were discovered in all MDRSP isolates. Pilus islet type 1 (PI-1) encoded by *rrgC* was found in 93 % (100/104) of MDRSP strains. Meanwhile, pilus type 2 (PI-2), which was detected by the presence of *pitB*, was observed in 82 % of MDRSP strains (89/104). The isolates that only carried PI-1 (14 %, 15/104) were higher in number than those that only carried PI-2 (4 %, 4/104). However, isolates of MDR serotypes 19F and 19A carrying both pili were also found among 79 % of isolates (85/104).

### Biofilm formation

Biofilm assay showed that 64 % (67/104) study isolates were not able to form biofilms or were non-producers. On the other hand, 34 % of MDRSP isolates serotypes 19F and 19A had moderate biofilm formation ability. In addition, one isolate was a strong biofilm producer.

### Sequence type

**Fig. 1.**
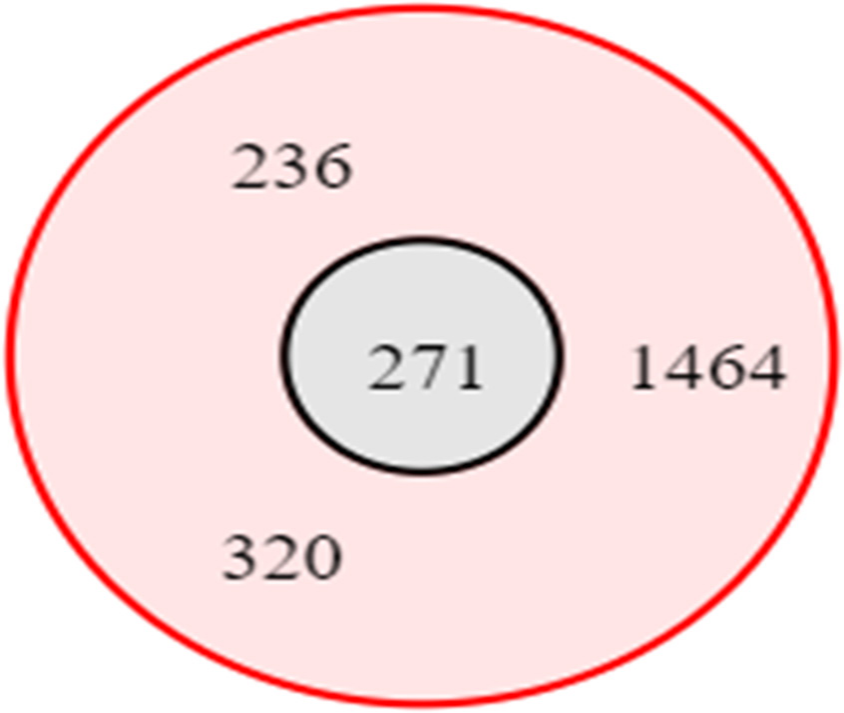
Analysis using burst showed ST271 as ancestral type.

Five out of 10 strains of MDRSP were ST1464 (Table 3). These five isolates were penicillin-resistant *S. pneumoniae*. Meanwhile, two isolates were ST320 (penicillin-resistant) and one isolate each was ST236 (penicillin-resistant), ST271 (penicillin-intermediate) and ST5047 (penicillin-susceptible). The difference in allele numbers in ST1464, ST320, ST236 and ST271 occurred mainly in the *ddl* gene. ST1464 and ST320 were *ddl* 104 and 1, respectively. ST236, ST271 and ST5047 were *ddl* 26. Although all four of these STs were included in *ddl* 26, ST271 and ST236 had differences in the *aroE* gene (*aroE* 4 for ST271 and *aroE* 15 for ST236). On the other hand, ST5047, which was also *ddl* 26, has quite a few differences in allele numbers, especially in the *gdh*, *recP* and *spi* genes. Analysis of STs using Based Upon Related Sequence Types (burst) showed that the potential ancestral type (AT) of the identified STs in this study was ST271 (Fig. 1). Meanwhile, ST5047 was only a singleton because the allelic profile was quite far from other STs.

**Table 3.** Sequence type from the combination of seven allele numbers of *S. pneumoniae* housekeeping genes in penicillin-resistant isolates

Specimen ID	Serotype	Allele no.							ST
		*aroE*	*gdh*	*gki*	*recP*	*spi*	*xpt*	*ddl*	
KBR0022	19F	4	16	19	15	6	20	106	1464
KBR0042	19F	4	16	19	15	6	20	26	271
KBR0062	19F	4	16	19	15	6	20	106	1464
KBR0083	19F	4	16	19	15	6	20	106	1464
WKT0041	19F	15	5	19	5	17	20	26	5047
2041 col 2	19F	4	16	19	15	6	20	1	320
2337	19F	4	16	19	15	6	20	106	1464
2350	19F	4	16	19	15	6	20	106	1464
2427	19F	15	16	19	15	6	20	26	271
2975	19A	4	16	19	15	6	20	1	320

## Discussion

This study showed that almost all the MDRSP serotype 19F and 19A isolates carried the *mefA* gene (98 %). Only half of the total isolates carried the *ermB* gene (52 %). In addition, the prevalence of isolates that carried only the *mefA* gene (44 %) was higher than that for isolates carrying the *ermB* gene only (3 %). The majority of the MDRSP isolates had MIC values of >2 µg ml^−1^ for erythromycin (89/93), with 58 % (52/89) of the isolates having *ermB,* 100 % encoded *mefA* and 55 % (42/89) both *ermB* and *mefA*. High-level macrolide resistance is generally associated with the *ermB* gene. The *mefA* gene causes lower macrolide resistance [24]. Study in Peru showed that the main macrolide resistance mechanism was mediated by *ermB* and mostly found in serotype 19A [25].

Most of the study isolates (103/104) also carried the *tetM* gene. The MIC value for tetracycline reached >8 µg ml^−1^, which might be due to the presence of the high proportion of *tetM* among the isolates. The main source of the *tetM* gene is the Tn*916* transposon [26]. The Tn*916* transposon also has resistance genes for tetracycline (*tetM* and *tetO*) and erythromycin (*ermB*). This can lead to pneumococcal isolates that are resistant to tetracycline to also be resistant to macrolides. This study has shown that macrolide-resistant *S. pneumoniae* can also be tetracycline-resistant. Our study showed a high proportion of *tetM* among MDRSP strains. This result is in concordance with the finding in Poland that tetracycline-resistant pneumococcus were dominated by serotypes 19F, 19A and 14 and the isolates possessed Tn*916* [27]. Moreover, high bacterial resistance to tetracycline may stem from overuse of the antibiotic tetracycline due to its accessibility and low cost [26].

MDRSP isolates serotypes 19F and 19A in Indonesia also have high resistance to co-trimoxazole. In this study, co-trimoxazole resistance was increasingly high, reaching 95 %. A carriage study performed in Lombok reported that 62 % of the pneumococcus isolated from healthy children <5 years were resistant to co-trimoxazole [9]. This co-trimoxazole resistance may be due to a mutation in the *folA* and *folP* genes encoding for DHFR and DHPS enzymes that are crucial for folic acid biosynthesis. Furthermore, co-trimoxazole is a widely used antibiotic, especially in resource-limited countries due to its low price, safe toxicity profile, availability for oral and intravenous routes, and its bactericidal activity [28]. Co-trimoxazole is also the second most frequently prescribed antibiotic for children in health facilities in Indonesia [29].

Insertion of arginine and proline (STRP ***RP*** GSSYVEIE) was the most frequent mutation in amino acids 56–67 of *folP*, followed by the insertion of 1 amino acid tyrosine (STRPGSSY ***Y*** VEIE), and 2 amino acids serine and tyrosine at the 63rd amino acid (STRPGSSY ***SY*** VEIE). Amino acids 58–67 comprise the binding site region for sulfamethoxazole on the DHPS enzyme [30]. Mutations at bases 58–67 were the most common mutations found in sulfamethoxazole-resistant *S. pneumoniae*. The insertion at the DHPS binding site results in a significant decrease in the enzyme’s affinity for sulfamethoxazole and causes the resistance of *S. pneumoniae* to sulfamethoxazole [6]. In addition, the insertion of arginine and proline at the 59th amino acid of DHPS changes the binding site of the sulfamethoxazole antibiotic to the DHPS enzyme, resulting in the resistance to sulfamethoxazole [31, 32].

Most of the MDRSP isolates had substitutions Asp-92–Ala, Glu-94–Asp and Ile-100–Leu in amino acids 92–100 of *folA*. The dominance of Asp-92–Ala, Glu-94–Asp and Ile-100–Leu was in line with a study conducted in Tanzania, which reported that the substitution combination of Asp-92–Ala, Glu-94–Asp and Ile-100–Leu was the most frequent substitution among co-trimoxazole-resistant *S. pneumoniae* isolates [33]. In addition, a study in Uganda showed that all co-trimoxazole-resistant *S. pneumoniae* isolates had the Ile-100–Leu mutation in *folA*. Meanwhile, a study in Malawi reported that 70 % of the trimethoprim-resistant *S. pneumoniae* isolates carried the Ile-100–Leu substitution [6]. Maskell *et al*. showed that trimethoprim resistance caused by Ile-100–Leu and Asp-92–Ala was accompanied by a significant decrease in the affinity of the enzyme to its natural substrate [34]. This indicated that trimethoprim resistance gained a considerable risk to enzyme function. Variations of Asp-92–Ala and Glu-94–Asp were also frequently found [17].

Ile-100–Leu substitution is important mutation in the development of trimethoprim resistance [6, 33]. Mutation in other locations increased the resistance against co-trimoxazole [6]. The combination of Ile-100–Leu and Asp-92–Ala was significant in causing trimethoprim resistance and increased the MIC value of 4 g ml^−1^ to 128 g ml^−1^ [34]. Ile-100–Leu substitution occurs at the trimethoprim substrate-binding site of the DHFR enzyme. Substitution of isoleucine with leucine decreases the affinity for trimethoprim in DHFR without affecting the binding of the dihydrofolate substrate. If the Ile-100–Leu mutation is transferred to a susceptible strain, it will cause the strain to become resistant [35].

On the other hand, the substitution combination of Asp-92–Ser, Glu-94–Asp and Ile-100–Leu was also found in this study (Table 2). Combinations of Asp-92–Ser with Glu-94–Asp and Ile-100–Leu were sufficient to create trimethoprim resistance with high MIC values [34]. Other substitutions that were identified among MDRSP isolates were a combination of Asp-92–Ala and Ile-100–Leu, and Asp-92–Ala alone. One of the study isolates that did not have mutations in Ile-100–Leu (only Asp-92–Ala) was a co-trimoxazole-susceptible isolate. This showed the importance of Ile-100–Leu in causing co-trimoxazole resistance. However, another isolate with a mutation in Asp-92–Ala but without a mutation in Ile-100–Leu is resistant to co-trimoxazole. This might be due to mutations in other regions of *folA* causing co-trimoxazole resistance. A study in Malawi also showed that the substitution Asp-92–Arg without the Ile-100–Leu mutation increased the MIC value of trimethoprim [6]. Further studies are thus needed to understand the effect of substitution without Ile-100–Leu on the changes in the binding site of DHFR leading to co-trimoxazole resistance.

All study isolates had pili, and almost all of them carried PI-1. In addition, the proportion of MDRSP isolates carrying both types of pili was high, reaching 82 %. Serotypes of *S. pneumoniae* that usually carry PI-1 were vaccine-covered serotypes such as 4, 6B, 9V, 14 and 19F [26]. PI-2 in serotypes 19F and 19A was in line with a report by Bagnoli *et al*. that PI-2 was associated with serotypes 1, 2, 7F, 19A and 19F [36]. However, the proportion of PI-2 was 10 % lower than for PI-1. In 2016, a pilus study in Iran showed that 40 % of carriage and invasive isolates of *S. pneumoniae* carried PI-1, but the PI-2 pilus was not found [18]. However, PI-2 was found in 21 % of invasive isolates in the USA and was mostly identified in serotypes 19F (40 %) and 7F (89 %) [37]. The distribution of PI-1 and PI-2 depends on the geographical regions and population.

More than 50 % of MDRSP serotypes 19F and 19A isolates could not form biofilm. One third of the isolates were moderate biofilm producers and one isolate was a strong biofilm producer. Biofilm formation ability was independent of whether they were vaccine serotypes or not [20]. Serotypes 6B, 15B/15C, 19A 35F and 35B were good biofilm-producing serotypes. Meanwhile, serotypes 23B, 23F and 19F had the lowest biofilm-producing ability. A lower proportion of biofilm producers among MDRSP might also be due to these strains having been isolated from healthy children. A biofilm-producing strain is usually found in *S. pneumoniae* causing acute otitis media. Vermee *et al*. reported that 67 % of *S. pneumoniae* from the nasopharynx of children with otitis media produced biofilm, with serotypes 6B, 15B/C, 19A, 35F and 35B being better biofilm producers [20]. Biofilm plays a crucial role in the pathophysiology of otitis media caused by *S. pneumoniae* [38]. Resistant isolates usually belong to a unique ST and are associated with resistant clones found in other countries.

The MLST test was carried out on 10 isolates that were resistant, intermediate and susceptible to penicillin. These 10 isolates were also resistant to macrolides, tetracyclines and co-trimoxazole. The results of the MLST test showed that the most identified STs were ST1464 (5/10), followed by ST320 (2/10), and ST236 (1/10), ST271 (1/10) and ST5047 (1/10). The predominance of ST1464 and ST271 was in line with the MLST study in the Czech Republic on penicillin-resistant isolates, which showed the predominance of the 19F, with the most identified STs being ST1464 and ST271 [39]. Analysis of penicillin-binding proteins (PBPs) also revealed that the PBP profiles of ST1464 and ST271 were similar to those of the Taiwan^19F-14^ [39].

The findings of ST1464, ST320, ST236 and ST271 in MDRSP isolates serotype 19F and 19A in Indonesia were in line with several studies on resistant *S. pneumoniae* isolates in Asia. The predominant STs of *S. pneumoniae* serotype 19F in children aged <5 years in the Republic of Korea were ST271 (21 %), ST236 (21 %), ST1464 (15 %), ST283 (10 %) and ST320 (9 %). Meanwhile, almost all isolates of serotype 19A were ST320 (90 %). The expansion of multidrug-resistant ST320 is responsible for the high prevalence of 19A before PCV7 vaccination in the Republic of Korea [40]. In addition, a 2017 study in PR China showed that 19A-ST320, 19F-ST271 and 14-ST876 led to high rates of resistance to macrolides, tetracyclines and penicillin in *S. pneumoniae* isolated from invasive sites [41]. ST236 also predominated in the majority of *S. pneumoniae* that were resistant to erythromycin, tetracycline and co-trimoxazole in Malaysia [42].

The results of this study showed the possession of antibiotic resistance genes and pili and a lack of biofilm formation among tested MDRSP serotype 19F and 19A isolates. This study, however, has its limitations. All of the tested isolates were from nasopharyngeal carriage that not describe the resistance or virulence factor of strain causing IPD. In addition, the samples used were uniform, with all being serotype 19F and 19A MDRSP strains. Therefore, the significant contribution of resistance genes, pili and biofilms to the development of resistance could not be calculated in comparison to non-MDRSP or other serotype strains. The MLST study was also only performed for 10 MDRSP isolates; a small sample size with less power for the description of the sequence types of MDRSP in Indonesia.

The results of this study are expected to provide additional information for the implementation of PCV13 vaccination in Indonesia as serotypes 19F and 19A are covered by PCV13 and tend to be more resistant than other serotypes. Moreover, information regarding the resistance genes among MDRSP is valuable for developing treatments for pneumococcal disease.

## Supplementary Data

Supplementary material 1

Supplementary material 2
