# Supplementary material for: Characterization of MultidrugResistant serogroup 19 Streptococcus pneumoniae isolated from healthy children below 5 years of age in Indonesia
Source: Access Microbiol. 2024 Feb 13;6(2):000680.v4. doi: 10.1099/acmi.0.000680.v4 (PMC10928408; doi:10.1099/acmi.0.000680.v4)
Supplement: Supplementary material 2 [file acmi-6-680.v4-s002.pdf]

Table S2. Primers and PCR Conditions for detecting resistance genes

No.	Gene	Primer sequence	Amplicon size (bp)	PCR condition	Reference
1	*ermB*	F: 5’-GAAAAGGTACTCAACCAAATA-3’ R: 5’-GTAACGGTACTTAAATTGTTTAC-3	639	Pre-denaturation at 94°C for 4 minutes. Followed by 35 cycles of: denaturation at 94°C for 30 seconds, annealing 50°C for 30 seconds, and elongation at 72°C for 1.5 minutes. Post-elongation at 72°C for 5 minutes	15
2	*mefA*	F: 5’-AGTATCATTAATCACTAGTGC-3’ R: 5’-TTCTTCTGGTACTAAAAGTGG-3’	348		
3	*tetM*	F: 5’-AGTGGAGCGATTACAGAA-3’ R: 5’-CATATGTCCTGGCGTGTCTA-3’	159	Pre-denaturation at 94°C for 4 minutes. Followed by 30 cycles of: denaturation at 94°C for 30 seconds, annealing 52°C for 30 seconds, and elongation at 72°C for 30 seconds. Post-elongation at 72°C for 7 minutes	16

4	*folA*	F: 5’-TGTAAGCTATTCCAAACCAG-3’ R: 5’-CTACGTTCCATTAGACTTCC-3’	600	Pre-denaturation at 94°C for 5 minutes. Followed by 35 cycles of: denaturation at 94°C for 1 minute, annealing 53°C for 1 minute, and elongation at 72°C for 30 seconds. Post-elongation at 72°C for 7 minutes	17
5	*folP*	F: 5’-GTCAAGTAAAGCCAATCATG-3’ R: 5’-AATTTTCCGCTTCATCAGC-3’	900	Pre-denaturation at 94°C for 5 minutes. Followed by 35 cycles of: denaturation at 94°C for 1 minute, annealing 51°C for 1 minute, and elongation at 72°C for 1 minute and 30 seconds. Post-elongation at 72°C for 7 minutes	
6	Pilus islet type 1 encoded by *rrgC*	F: 5’-AACAGCCTGCTGGTTATGC-3’ R: 5’-TAGAGCGAACATAGTAAAGAC-3’	600	Pre-denaturation at 96°C for 4 minutes. Followed by 35 cycles of: denaturation at 94°C for 30 seconds, annealing 47°C for 30 seconds, and elongation at 72°C for 1 minute and 30 seconds. Post-elongation at 72°C for 10 minutes	18,19
7	Pilus islet type 2 encoded by *pitB*	F: 5’GAGTGTCTGGGGAGAATTCCTTTAC-3’ R: 5’-GGTTATTGCTGAATTAGGATCCGC-3’	600		
